# 2-(Phenyl­carbonothio­ylsulfan­yl)acetic acid

**DOI:** 10.1107/S160053681003686X

**Published:** 2010-09-25

**Authors:** Rodolfo Moreno-Fuquen, Carlos Grande, Fabio Zuluaga, Javier Ellena, Carlos A. De Simone

**Affiliations:** a Departamento de Química – Facultad de Ciencias, Universidad del Valle, Apartado 25360, Santiago de Cali, Colombia; b Instituto de Física, IFSC, Universidade de São Paulo, São Carlos, Brazil

## Abstract

The title compound, C_9_H_8_O_2_S_2_, can be used as a chain transfer agent and may be used to control the behavior of polymerization reactions. O—H⋯O hydrogen bonds of moderate character link the mol­ecules into dimers. In the crystal, the dimers are linked into sheets by C—H⋯O inter­actions, forming *R*_4_^2^(12) and *R*_2_^2^(8) edge-fused rings running parallel to [101]. There are no inter­molecular inter­actions involving the S atoms.

## Related literature

For the use of dithio­carbonyl components as chain transfer agents in polymerization reactions, see: Mayadunne *et al.* (1999 ▶); Davis (2004 ▶). For related structures, see: Adiwidjaja & Voss (1977 ▶); Liang *et al.* (2008 ▶). For hydrogen bonding, see: Etter (1990 ▶); Nardelli (1995 ▶); Emsley (1984 ▶). For a description of the Cambridge Structural Database, see: Allen (2002 ▶).
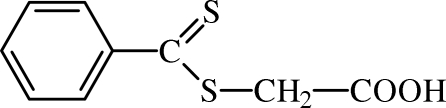


## Experimental

### 

#### Crystal data

C_9_H_8_O_2_S_2_
*M* *_r_* = 212.29
Monoclinic, 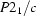

*a* = 13.1565 (7) Å
*b* = 4.9522 (2) Å
*c* = 17.3747 (7) Å
β = 121.870 (3)°
*V* = 961.37 (8) Å^3^
*Z* = 4
Mo *K*α radiation
μ = 0.52 mm^−1^
*T* = 291 K
0.26 × 0.22 × 0.16 mm

#### Data collection

Bruker–Nonius KappaCCD diffractometer
Absorption correction: multi-scan (*SADABS*; Sheldrick, 1996 ▶) *T*_min_ = 0.863, *T*_max_ = 0.916
5647 measured reflections
2131 independent reflections
1682 reflections with *I* > 2σ(*I*)
*R*_int_ = 0.063

#### Refinement

*R*[*F* ^2^ > 2σ(*F* ^2^)] = 0.047
*wR*(*F* ^2^) = 0.148
*S* = 1.05
2131 reflections
118 parameters
H-atom parameters constrained
Δρ_max_ = 0.23 e Å^−3^
Δρ_min_ = −0.42 e Å^−3^

### 

Data collection: *COLLECT* (Nonius, 1998 ▶); cell refinement: *SCALEPACK* (Otwinowski & Minor, 1997 ▶); data reduction: *DENZO* (Otwinowski & Minor, 1997 ▶) and *SCALEPACK*; program(s) used to solve structure: *SHELXS97* (Sheldrick, 2008 ▶); program(s) used to refine structure: *SHELXL97* (Sheldrick, 2008 ▶); molecular graphics: *ORTEP-3 for Windows* (Farrugia, 1997 ▶); software used to prepare material for publication: *WinGX* (Farrugia, 1999 ▶).

## Supplementary Material

Crystal structure: contains datablocks I, global. DOI: 10.1107/S160053681003686X/jh2203sup1.cif

Structure factors: contains datablocks I. DOI: 10.1107/S160053681003686X/jh2203Isup2.hkl

Additional supplementary materials:  crystallographic information; 3D view; checkCIF report

## Figures and Tables

**Table 1:** Hydrogen-bond geometry (Å, °)

*D*—H⋯*A*	*D*—H	H⋯*A*	*D*⋯*A*	*D*—H⋯*A*
O2—H22⋯O1^i^	0.82	1.85	2.658 (2)	167
C8—H8*B*⋯O1^ii^	0.97	2.63	3.439 (3)	141

Symmetry codes: (i) 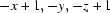; (ii) 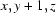.

**Fig. 1.**
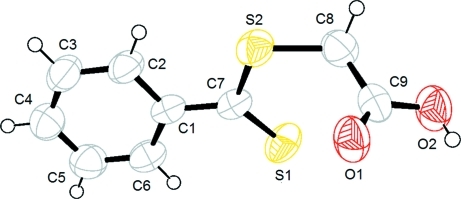
An ORTEP-3 (Farrugia, 1997) plot of the title compound with the atomic labelling scheme. Displacement ellipsoids are drawn at the 50% probability level. H atoms are shown as spheres of arbitary radius.

**Fig. 2.**
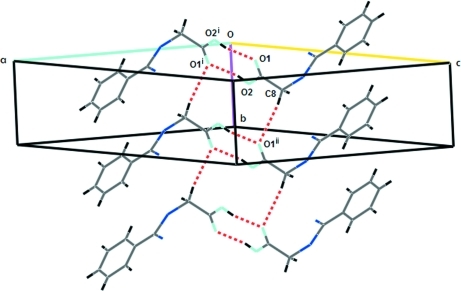
Part of the crystal structure of (I), showing the formation of R42(12) and R22(8) running parallel to the [101] direction. Symmetry code: (i)-x + 1, -y, -z + 1; (ii) x, y + 1, z.

**Fig. 3.**
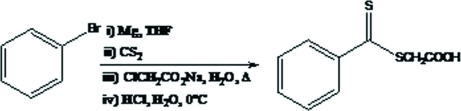
The foramtion of the title compound.

## supplementary crystallographic information

### Comment

Controlled behavior in a polymerization reaction can be achieved with the presence of a dithiocarbonyl component, used as a chain transfer agent (CTA). These agents have the ability to react by changing the activity of a growing polymer to another molecule, producing new polymer chains (Mayadunne *et al.*, 1999; Davis, 2004) and reducing the average molecular weight at the final polymer. Continuing research on these materials, the Polymer group of the Universidad of Valle, synthesized the 2-(phenylcarbonothioylthio) acetic acid molecule. A displacement ellipsoid plot of the title molecule with the atomic numbering scheme is shown in Figure 1. Carboxylic acids usually exist as dimeric pairs. Indeed, a hydrogen bond of moderate character (Emsley, 1984) between the O2 atom at (*x*,*y*,*z*) and the O1 atom at (1 - *x*,-*y*, 1 - *z*) in the title molecular complex is observed. The O2···O1 distance is 2.658 (3) Å and the O2—H2···O1 angle is 167.1 (2)°. The dimers of the title molecule, are linked into sheets by a weak C—H···O intermolecular interactions (Table 1)(Nardelli, 1995). Indeed, the C8 atom at (*x*,*y*,*z*) acts as hydrogen bond donor to carboxyl O1 atom in the molecule at (*x*, *y* + 1,*z*) forming *R*_4_^2^(12) and *R*_2_^2^(8) edge-fused rings (Etter, 1990) running parallel to the [101] direction (see Fig 2). The title compound shows a C1=S7 distance of 1.6319 (19) Å suggesting a double-bond character and C2—S7 and C2—S8 distances of 1.745 (2) and 1.786 (2) Å respectivelly, suggesting a single bond character. A dihedral angle of 12.37 (12)° between the plane formed by the atoms C8/S2/C7/S1 and the plane of benzene is observed. The behavior of these bond lengths is similar to that observed in the Methyl 4 - t-butyldithiobenzoate and methylene bis(dithiobenzoate) structures (Adiwidjaja & Voss, 1977; Liang *et al.*, 2008). There are no intermolecular interactions from S atoms.

### Experimental

Synthesis of 2-(phenylcarbonothioylthio)acetic acid: 12.56 g (0.08 mol) of bromobenzene was added dropwise to a solution of 50 ml of dry THF, 2.00 g (0.08 mol) of magnesium stirrings and a crystal of iodine. Once the reaction was finished, 6.09 g (0.08 mol) of CS2 were added and a dark violet solution was obtained after stirring for 2 h at room temperature. Then, a solution of 7.56 g (0.08 mol) of chloroacetic acid in 200 ml of water, was prepared and neutralized with 6.72 g (0.08 mol) of solid sodium bicarbonate, which was rapidly added through the condenser, the mixture was stirred, brought to boiling and left refluxing for 5 minutes. The resulting brownish red suspension was added to 500 g of cold water and the resulting solution was slowly acidified under stirring with concentrated hydrochloric acid. A deep-scarlet crystalline precipitate was collected after 30 minutes at 0°C, rinsed with water and then crystallized in chloroform obtaining a red solid (9.17 g, 54% yield).

^1^H (400 MHz) Solvent: CDCl3 NMR (p.p.m.) δ: 4.30 (s, 2 H, –CH2), 7.43 (t, 2H, *m*-ArH, J = 8 Hz) 7.59 (t, 1H, *p*-ArH, J = 8 Hz), 8.07 (d, 2H, *o*-ArH, J = 8 Hz), 9.14 (s, 1H, –OH).

IR: (KBr) 3200–2800 –COOH; 3000–2850, –CH; 1700, C=O; 1050, C=S.

### Refinement

All non-hydrogen atoms were identified by direct methods. The H atoms were all located in a difference map, but those attached to carbon atoms were repositioned geometrically. The H atoms were initially refined with soft restraints on the bond lengths and angles to regularize their geometry. (C—H in the range 0.93–0.97 A°) and *U*_iso_(H) (in the range 1.2–1.5 times *U*_eq_ of the parent atom). After this, the positions were refined with riding constraints.

### Crystal data

**Table:**

C_9_H_8_O_2_S_2_	*F*(000) = 440
*M**_r_* = 212.29	*D*_x_ = 1.467 Mg m^−^^3^
Monoclinic, *P*2_1_/*c*	Melting point: 399(1) K
Hall symbol: -P 2ybc	Mo *K*α radiation, λ = 0.71073 Å
*a* = 13.1565 (7) Å	Cell parameters from 3433 reflections
*b* = 4.9522 (2) Å	θ = 2.9–27.5°
*c* = 17.3747 (7) Å	µ = 0.52 mm^−^^1^
β = 121.870 (3)°	*T* = 291 K
*V* = 961.37 (8) Å^3^	Prism, red
*Z* = 4	0.26 × 0.22 × 0.16 mm

### Data collection

**Table:**

Bruker–Nonius KappaCCD diffractometer	2131 independent reflections
Radiation source: fine-focus sealed tube	1682 reflections with *I* > 2σ(*I*)
horizonally mounted graphite crystal	*R*_int_ = 0.063
Detector resolution: 9 pixels mm^-1^	θ_max_ = 27.5°, θ_min_ = 3.2°
CCD scans	*h* = −17→16
Absorption correction: multi-scan (*SADABS*; Sheldrick, 1996)	*k* = −5→6
*T*_min_ = 0.863, *T*_max_ = 0.916	*l* = −22→17
5647 measured reflections	

### Refinement

**Table:**

Refinement on *F*^2^	Primary atom site location: structure-invariant direct methods
Least-squares matrix: full	Secondary atom site location: difference Fourier map
*R*[*F*^2^ > 2σ(*F*^2^)] = 0.047	Hydrogen site location: inferred from neighbouring sites
*wR*(*F*^2^) = 0.148	H-atom parameters constrained
*S* = 1.05	*w* = 1/[σ^2^(*F*_o_^2^) + (0.0898*P*)^2^ + 0.0835*P*] where *P* = (*F*_o_^2^ + 2*F*_c_^2^)/3
2131 reflections	(Δ/σ)_max_ < 0.001
118 parameters	Δρ_max_ = 0.23 e Å^−^^3^
0 restraints	Δρ_min_ = −0.42 e Å^−^^3^

### Special details

**Table:**

Geometry. All e.s.d.'s (except the e.s.d. in the dihedral angle between two l.s. planes) are estimated using the full covariance matrix. The cell e.s.d.'s are taken into account individually in the estimation of e.s.d.'s in distances, angles and torsion angles; correlations between e.s.d.'s in cell parameters are only used when they are defined by crystal symmetry. An approximate (isotropic) treatment of cell e.s.d.'s is used for estimating e.s.d.'s involving l.s. planes.
Refinement. Refinement of *F*^2^ against ALL reflections. The weighted *R*-factor *wR* and goodness of fit *S* are based on *F*^2^, conventional *R*-factors *R* are based on *F*, with *F* set to zero for negative *F*^2^. The threshold expression of *F*^2^ > σ(*F*^2^) is used only for calculating *R*-factors(gt) *etc*. and is not relevant to the choice of reflections for refinement. *R*-factors based on *F*^2^ are statistically about twice as large as those based on *F*, and *R*- factors based on ALL data will be even larger.

### Fractional atomic coordinates and isotropic or equivalent isotropic displacement parameters (Å^2^)

**Table:**

	*x*	*y*	*z*	*U*_iso_*/*U*_eq_	
S2	0.36260 (5)	0.16659 (12)	0.68630 (3)	0.0659 (2)	
S1	0.15902 (5)	−0.03907 (14)	0.51228 (4)	0.0750 (3)	
O1	0.47853 (15)	−0.0386 (3)	0.58577 (12)	0.0719 (4)	
C1	0.19825 (17)	−0.1776 (3)	0.67884 (13)	0.0517 (4)	
O2	0.40702 (16)	0.2866 (3)	0.48242 (12)	0.0784 (5)	
H22	0.4350	0.1905	0.4597	0.118*	
C7	0.23335 (16)	−0.0269 (4)	0.62278 (13)	0.0528 (4)	
C6	0.11223 (19)	−0.3787 (5)	0.64001 (15)	0.0647 (5)	
H6	0.0759	−0.4163	0.5785	0.078*	
C9	0.42419 (18)	0.1733 (4)	0.55576 (14)	0.0576 (5)	
C8	0.3752 (2)	0.3384 (4)	0.60136 (17)	0.0686 (6)	
H8A	0.2966	0.4038	0.5554	0.082*	
H8B	0.4264	0.4947	0.6289	0.082*	
C2	0.2493 (2)	−0.1244 (5)	0.77093 (13)	0.0637 (5)	
H2	0.3063	0.0116	0.7982	0.076*	
C5	0.0800 (2)	−0.5241 (5)	0.69193 (19)	0.0737 (6)	
H5	0.0228	−0.6599	0.6651	0.088*	
C3	0.2166 (2)	−0.2708 (5)	0.82243 (15)	0.0722 (6)	
H3	0.2521	−0.2345	0.8839	0.087*	
C4	0.1313 (2)	−0.4702 (5)	0.78224 (19)	0.0737 (6)	
H4	0.1086	−0.5681	0.8165	0.088*	

### Atomic displacement parameters (Å^2^)

**Table:**

	*U*^11^	*U*^22^	*U*^33^	*U*^12^	*U*^13^	*U*^23^
S2	0.0613 (4)	0.0789 (4)	0.0479 (3)	−0.0101 (2)	0.0221 (3)	−0.0074 (2)
S1	0.0703 (4)	0.1042 (5)	0.0390 (3)	−0.0052 (3)	0.0210 (3)	−0.0069 (2)
O1	0.0796 (10)	0.0692 (9)	0.0760 (11)	0.0162 (8)	0.0472 (9)	0.0244 (7)
C1	0.0490 (9)	0.0562 (9)	0.0446 (9)	0.0059 (7)	0.0210 (8)	−0.0078 (7)
O2	0.0974 (12)	0.0695 (9)	0.0792 (11)	0.0184 (8)	0.0542 (10)	0.0278 (8)
C7	0.0506 (9)	0.0583 (10)	0.0434 (9)	0.0086 (8)	0.0207 (8)	−0.0054 (7)
C6	0.0602 (11)	0.0739 (12)	0.0511 (11)	−0.0039 (10)	0.0233 (9)	−0.0136 (9)
C9	0.0550 (10)	0.0556 (10)	0.0573 (11)	−0.0045 (8)	0.0263 (9)	0.0085 (8)
C8	0.0771 (14)	0.0578 (11)	0.0687 (13)	−0.0022 (10)	0.0370 (12)	0.0027 (9)
C2	0.0667 (12)	0.0718 (12)	0.0462 (10)	−0.0068 (10)	0.0255 (9)	−0.0115 (9)
C5	0.0754 (14)	0.0721 (13)	0.0770 (16)	−0.0107 (11)	0.0425 (13)	−0.0106 (11)
C3	0.0836 (15)	0.0831 (14)	0.0525 (12)	0.0008 (13)	0.0378 (11)	−0.0039 (11)
C4	0.0866 (15)	0.0719 (13)	0.0762 (16)	0.0031 (11)	0.0521 (14)	0.0021 (11)

### Geometric parameters (Å, °)

**Table:**

S2—C7	1.745 (2)	C9—C8	1.500 (3)
S2—C8	1.786 (2)	C8—H8A	0.9700
S1—C7	1.6319 (19)	C8—H8B	0.9700
O1—C9	1.221 (2)	C2—C3	1.385 (3)
C1—C6	1.386 (3)	C2—H2	0.9300
C1—C2	1.395 (3)	C5—C4	1.368 (4)
C1—C7	1.481 (3)	C5—H5	0.9300
O2—C9	1.298 (2)	C3—C4	1.376 (4)
O2—H22	0.8200	C3—H3	0.9300
C6—C5	1.385 (3)	C4—H4	0.9300
C6—H6	0.9300		
			
C7—S2—C8	102.92 (10)	S2—C8—H8A	108.4
C6—C1—C2	118.0 (2)	C9—C8—H8B	108.4
C6—C1—C7	119.99 (18)	S2—C8—H8B	108.4
C2—C1—C7	122.03 (18)	H8A—C8—H8B	107.5
C9—O2—H22	109.5	C3—C2—C1	121.1 (2)
C1—C7—S1	123.62 (15)	C3—C2—H2	119.5
C1—C7—S2	113.50 (14)	C1—C2—H2	119.5
S1—C7—S2	122.88 (12)	C4—C5—C6	120.7 (2)
C5—C6—C1	120.6 (2)	C4—C5—H5	119.7
C5—C6—H6	119.7	C6—C5—H5	119.7
C1—C6—H6	119.7	C4—C3—C2	119.7 (2)
O1—C9—O2	123.3 (2)	C4—C3—H3	120.1
O1—C9—C8	124.10 (19)	C2—C3—H3	120.1
O2—C9—C8	112.49 (17)	C5—C4—C3	119.9 (2)
C9—C8—S2	115.50 (14)	C5—C4—H4	120.0
C9—C8—H8A	108.4	C3—C4—H4	120.0
			
C6—C1—C7—S1	12.5 (2)	O2—C9—C8—S2	165.81 (16)
C2—C1—C7—S1	−167.52 (16)	C7—S2—C8—C9	−78.01 (18)
C6—C1—C7—S2	−167.49 (14)	C6—C1—C2—C3	1.0 (3)
C2—C1—C7—S2	12.5 (2)	C7—C1—C2—C3	−178.9 (2)
C8—S2—C7—C1	−176.11 (13)	C1—C6—C5—C4	0.7 (4)
C8—S2—C7—S1	3.88 (15)	C1—C2—C3—C4	−0.8 (4)
C2—C1—C6—C5	−1.0 (3)	C6—C5—C4—C3	−0.5 (4)
C7—C1—C6—C5	178.96 (19)	C2—C3—C4—C5	0.5 (4)
O1—C9—C8—S2	−17.0 (3)		

### Hydrogen-bond geometry (Å, °)

**Table:**

*D*—H···*A*	*D*—H	H···*A*	*D*···*A*	*D*—H···*A*
O2—H22···O1^i^	0.82	1.85	2.658 (2)	167
C8—H8B···O1^ii^	0.97	2.63	3.439 (3)	141

Symmetry codes: (i) −*x*+1, −*y*, −*z*+1; (ii) *x*, *y*+1, *z*.
